# The *Arabidopsis thaliana-Alternaria brassicicola* pathosystem: A model interaction for investigating seed transmission of necrotrophic fungi

**DOI:** 10.1186/1746-4811-8-16

**Published:** 2012-05-09

**Authors:** Stephanie Pochon, Emmanuel Terrasson, Thomas Guillemette, Beatrice Iacomi-Vasilescu, Sonia Georgeault, Marjorie Juchaux, Romain Berruyer, Isabelle Debeaujon, Philippe Simoneau, Claire Campion

**Affiliations:** 1 Université d’Angers, UMR 1345 IRHS, SFR QUASAV, 2 Bd Lavoisier, Angers cedex, F-49045, France; 2 INRA, UMR 1345 IRHS, 16 Bd Lavoisier, Angers cedex, F-49045, France; 3 Agrocampus-Ouest, UMR 1345 IRHS, 2 Bd Lavoisier, Angers cedex, F-49045, France; 4 USAMV, 59 Bd Marasti, Bucharest, Ro-71331, Romania; 5 Université d’Angers, SCIAM, IBS, 4 rue Larrey, Angers cedex, F-49933, France; 6 Université d’Angers, SFR QUASAV, IMAC, rue Georges Morel, Beaucouzé cedex, F-49071, France; 7 INRA, UMR1318 IJPB, Saclay Plant Sciences, Route de Saint-Cyr, Versailles Cedex, 78026, France

**Keywords:** Seed transmission, *Alternaria brassicicola*, *Arabidopsis thaliana*, Seed colonization, *Transparent testa*, Osmotic stress

## Abstract

**Background:**

Seed transmission constitutes a major component of the parasitic cycle for several fungal pathogens. However, very little is known concerning fungal or plant genetic factors that impact seed transmission and mechanisms underlying this key biological trait have yet to be clarified. Such lack of available data could be probably explained by the absence of suitable model pathosystem to study plant-fungus interactions during the plant reproductive phase.

**Results:**

Here we report on setting up a new pathosystem that could facilitate the study of fungal seed transmission. Reproductive organs of *Arabidopsis thaliana* were inoculated with *Alternaria brassicicola* conidia. Parameters (floral vs fruit route, seed collection date, plant and silique developmental stages) that could influence the seed transmission efficiency were tested to define optimal seed infection conditions. Microscopic observations revealed that the fungus penetrates siliques through cellular junctions, replum and stomata, and into seed coats either directly or through cracks. The ability of the osmosensitive fungal mutant *nik1Δ3* to transmit to *A. thaliana* seeds was analyzed. A significant decrease in seed transmission rate was observed compared to the wild-type parental strain, confirming that a functional osmoregulation pathway is required for efficient seed transmission of the fungus. Similarly, to test the role of flavonoids in seed coat protection against pathogens, a *transparent testa Arabidopsis* mutant (*tt4-1*) not producing any flavonoid was used as host plant. Unexpectedly, *tt4-1* seeds were infected to a significantly lower extent than wild-type seeds, possibly due to over-accumulation of other antimicrobial metabolites.

**Conclusions:**

The *Arabidopsis thaliana-Alternaria brassicicola* pathosystem, that have been widely used to study plant-pathogen interactions during the vegetative phase, also proved to constitute a suitable model pathosystem for detailed analysis of plant-pathogen interactions during the reproductive phase. We demonstrated that it provides an excellent system for investigating the impact of different fungal or plant mutations on the seed transmission process and therefore paves the way towards future high-throughput screening of both *Arabidopsis* and fungal mutant.

## Background

Transmission from infected to healthy hosts is a key component of pathogen fitness, as pathogens strongly depend on this process for their long-term survival [1]. For several plant-parasite interactions, transmission is strictly “horizontal” with a pathogen spreading between related and unrelated individuals through mechanisms ranging from direct contact to wind-dispersal. In other systems, transmission may also be “vertical”, that is to say the pathogen infects the host’s offspring [2]. Although many pathogens spread by a combination of horizontal and vertical transmission, these two transmission modes require specific adaptations of the pathogen that can be mutually exclusive, resulting in a trade-off between horizontal and vertical transmission [1]. While an inverse relation between aggressiveness and vertical transmission has been shown for some pathogens [3], the ability to transmit by seed may have significant advantages for pathogens, such as long-term survival, maximum opportunity for progeny infection and long distance dissemination [4,5].

From an epidemiological standpoint, seed transmission represents a possible mode of primary infection from which dangerous polycyclic microorganisms can start epidemic attacks. The early presence of a small quantity of infected plants (i.e. 0.05% of a crop) is sometimes sufficient for the development of destructive attacks [6]. The use of seeds infected with pathogenic fungi thus represents a major economic threat and prevention of plant infection by seedborne pathogens in seed crops is essential. Although the chain of events leading to seed transfer through the plant has been extensively documented [4], almost nothing is currently known concerning the factors that mediate seed transmission by fungal pathogens. Iacomi-Vasilescu et al. [7] recently reported that *Alternaria brassicicola* strains deficient in a group III osmosensor histidine kinase were highly jeopardized in their ability to infect radish seeds. This was interpreted as a consequence of their failure to overcome severe osmotic stress conditions consecutive to the gradual decrease in the water potential in maturing seeds. Besides this pioneer study, no other data are available for fungal pathogenic factors that impact seed transmission. This could be probably explained by the difficulty to carry out such experiments under field conditions, particularly due to the reduced number of reproduction cycles per year and the lack of available model pathosystem to study plant-fungus interactions during the plant reproductive phase.

*Arabidopsis thaliana* has become an important model host for studying plant-pathogen interactions during the vegetative phase due to the development of numerous genetic and genomic tools. However, to our knowledge, this plant species has never been used as host to explore plant-pathogen interactions during the reproductive phase although it has several attributes such as small size and rapid life cycle, which make it well suited for this kind of study under carefully controlled environmental conditions. Among the numerous *Arabidopsis* – pathogen systems that have been developed to investigate the molecular mechanisms of virulence and plant resistance [8], the interaction of *Arabidopsis* with *Alternaria brassicicola* has been extensively used as a model for diseases caused by fungal necrotrophs [9]. *Alternaria brassicicola* causes dark spot disease, one of the most common and destructive fungal diseases of *Brassicaceae* worldwide. This fungus can be seedborne via mycelium within the seed and is the dominating *Alternaria* spp. in *Brassica* seed crops and may be responsible for important yield losses [10-13]. In such crops, a vertical disease gradient can be observed on fruits, with the lowest siliques becoming infected first and infection spreading slowly upwards [14]. Concerning seeds, hilum was described to be the more contaminated seed area, as a consequence of mycelial growth through the suture of siliques and the funicle [15,16]. To a lesser extent, *A. brassicicola* can infect seeds in an internal manner. In this instance, the pathogen is mainly found in seed coats. Both external and internal inoculum can survive for several years but the longevity of internal inoculum is higher than that of external inoculum [10].

The main objective of the present study was to develop, in controlled conditions, a model pathosystem suitable for detailed analysis of the molecular mechanisms underlying the seed transmission stage in both the host plant and the fungal pathogen. Reproductive organs of *A. thaliana* Landsberg *erecta* (L*er*), an ecotype susceptible to *A. brassicicola*, were thus inoculated with fungal conidia, and transmission levels of this pathogen to seeds were evaluated. The influence of different parameters on the seed transmission “probability” was established in order to design a robust and easy to handle protocol. This model pathosystem was then used to confirm that an *A. brassicicola* strain deficient in the osmosensing histidine kinase AbNIK1 was impaired in its capacity to efficiently colonize seeds. In addition, transmission of *A. brassicicola* to seeds of an *A. thaliana transparent testa 4* (*tt4-1*) mutant deficient with respect to flavonoid synthesis was also analyzed.

## Results

### Seed transmission route and influence of the seed collection date on seed transmission

As seed infection through the vascular supply has been described for wilt pathogens [17], only floral and fruit routes of seed infection were explored with *Alternaria brassicicola*. The floral infection route for seed transmission was studied in plants at the bolting stage by inoculating floral buds with an *A. brassicicola* (Abra43, WT) conidial suspension adjusted to either 1 x 10^4^ or 1 x 10^5^ conidia mL^-1^. For inoculations, 5 μl drops of an *A. brassicicola* conidial suspension were deposited on intact host tissue. Six days after inoculation, necrotic lesions were observed on flower buds and 8 days later, flower buds were covered with mycelium and conidia, irrespective of the inoculum charge. As a consequence, flower formation was impeded (data not shown).

**Figure 1:**
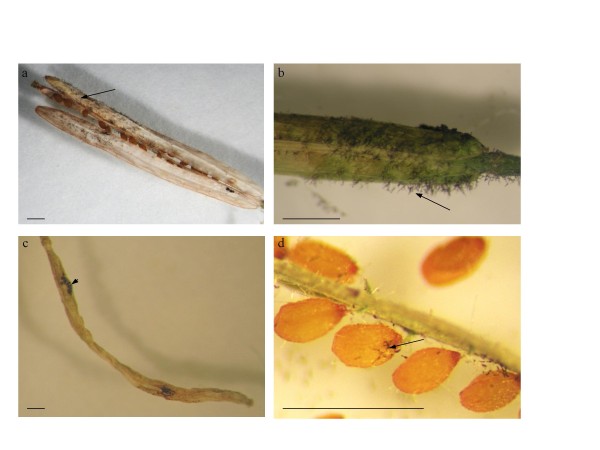
**Mycelium development, conidia formation on siliques and seeds, and necrotic lesions on siliques 10 days after inoculation with *Alternaria brassicicola*.** (**a**) and (**b**), mycelium development and conidia formation (arrows) on siliques. (**c**), necrotic lesions (arrowhead) on siliques. (**d**), mycelium development and conidia formation (arrow) on seeds. Bar = 1 mm.

**Figure 2:**
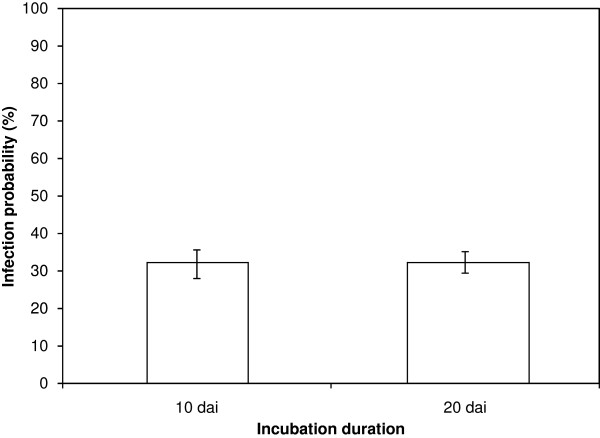
**Influence of incubation duration on transmission capacity of *Alternaria brassicicola *(Abra43) to seeds of *Arabidopsis thaliana *plants (L*er *ecotype).** Harvesting was done 10 days and 20 days after inoculation (dai). Values represent means with IC_95._

For examination of the fruit route for seed transmission, plants with 10 siliques on the main flowering stem, were inoculated by application of two distinct 2.5 μL drops of an *A. brassicicola* (Abra43) conidial suspension adjusted to 1 x 10^5^ conidia mL^-1^ on the outer surface of the 10 oldest siliques (one drop at the base of silique and one in the middle). Inoculations resulted in mycelium development, conidia formation and the appearance of necrotic lesions typical of black spot on siliques within a few days after inoculation (Figure 1a, 1b and 1c). Inoculated siliques were collected 10 or 20 days after inoculation, and seeds were individually harvested. Seeds showed mycelium development and conidia formation (Figure 1d). They were separately plated on PDA medium, to estimate the seed contamination level. Transmission of *A. brassicicola* to seeds was similar (32% of contaminated seeds) at the two collection dates (Figure 2). Siliques and seeds were thus harvested 10 days after inoculation for further experiments, since longer incubation did not induce a higher contamination level. Seeds harvested in control plants that were integrated to all experiments, and whose siliques were treated with two 2.5 μL drops of a 0.01% Tween 20 solution, never showed any *A. brassicicola* development.

### Influence of the plant and silique developmental stage on seed transmission

**Figure 3:**
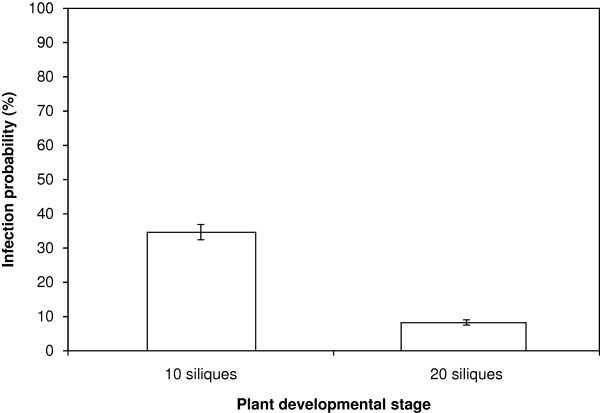
**Influence of plant developmental stage on transmission capacity of *Alternaria brassicicola *(Abra43) to seeds of *Arabidopsis thaliana* plants (L*er *ecotype).** Siliques were inoculated when 10 or 20 siliques were present on the main flowering stem. Harvesting was done 10 days after inoculation. Values represent means with IC_95._

The influence of the plant developmental stage on the incidence of *A. brassicicola* on seeds was examined by inoculating all siliques of plants with either 10 or 20 siliques on the main flowering stem. For inoculations, two distinct 2.5 μL drops of a conidial suspension adjusted to 1 x 10^5^ conidia mL^-1^ were applied. The results indicated that the contamination level per plant was significantly higher (35%) when siliques were inoculated at the 10-silique stage rather than later at the 20-silique stage (8%) (Figure 3). Further experiments were thus conducted by inoculating plants at the 10-silique stage.

**Figure 4:**
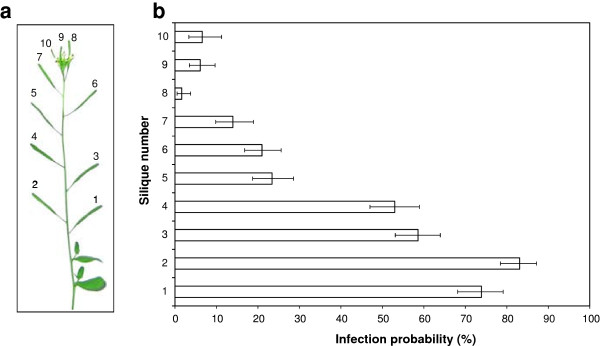
**Influence of silique stage on transmission capacity of *Alternaria brassicicola* (Abra43) to seeds of *Arabidopsis thaliana* plants (L*er *ecotype).** (**a**) Representation of the silique nomenclature. (**b**) Seed transmission level according to silique stage. Harvesting was done 10 days after inoculation. Values represent means with IC_95._

The impact of the silique developmental stage on *A. brassicicola* seed transmission was assessed through the inoculation of 10 siliques numbered from 1 (the oldest silique, at the base of the bolting stem) to 10 (the youngest silique) (Figure 4a), and the seeds were identified according to their corresponding silique. The global probability of seed infection by *A. brassicicola* was similar to previous results (32%, data not shown), but the seed infection probability differed depending on the silique: 74% for seeds from the oldest silique at the base of the bolting stem (n°1), only 7% for seeds from a younger silique near the apical meristem (n°10) (Figure 4b). The incidence on seeds was thus significantly different according to the silique developmental stage. As the *A. brassicicola* incidence on seeds of the youngest siliques was very low, in further experiments, evaluation of *A. brassicicola* seed transmission capacity took only the results concerning the five oldest siliques (n°1 to n°5) into account.

### Microscopic assessment of seed and silique colonization by *A. brassicicola*

Siliques and seeds harvested 10 days after inoculation of siliques with an *A. brassicicola* conidial suspension adjusted to 1 x 10^5^ conidia mL^-1^ were assessed by scanning electron microscopy (SEM) and confocal laser scanning microscopy. These studies focused on the histopathology of infected seeds and siliques, while observing the extent and amount of mycelium and conidia.

**Figure 5:**
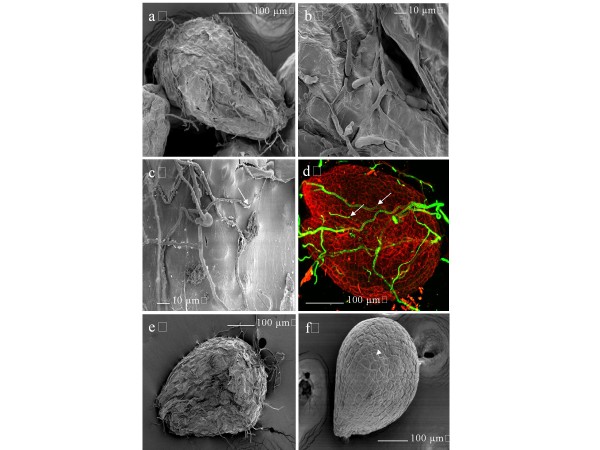
***A. brassicicola* localization on *A. thaliana* seeds.** Scanning electron microscopy a, b, c, e and **f**) and confocal laser scanning microscopy (**d**) images of *Arabidopsis thaliana* seeds (L*er* ecotype) 10 days after inoculation with *Alternaria brassicicola* (Abra43). (**a**) Global colonization of a seed by *A. brassicicola*. (**b**) conidia formation on a seed. (**c**) direct hyphal penetration into the seed coat (white arrow). (**d**) mycelium development on a seed. Hyphae were stained with solophenyl flavin, and seed coat with propidium iodide. Hyphae grew on the seed surface, then penetrated into the seed coat (white arrows): hyphal brightness was lower when hyphae were growing within the seed coat. (**e**) and (**f**)**,** alteration of the seed surface after fungal colonization: seed seemed shrivelled (**e**) by comparison with a seed harvested on a control plant (**f**).

SEM observations of seeds provided evidence of *A. brassicicola* mycelium superficial growth and conidia formation on seeds harvested 10 days after inoculation (Figure 5a and 5b). Penetration of hyphae into seed coat tissues, directly (Figure 5c) or through cracks in the seed coat, was observed by SEM. Penetration and fungal development within the seed tissues were also revealed by staining hyphae with solophenyl flavin and observing the tissues under a confocal laser scanning microscope (Figure 5d). These findings indicated that the fungus actively colonized the seed coat and was not only a passive contaminant of the seed surface. After fungal colonization, infected seeds frequently showed alteration of the seed coat appearance (Figure 5e) by comparison with seeds harvested on control plants (Figure 5f): infected seeds seemed shrivelled as if embryo aborted early during development.

**Figure 6:**
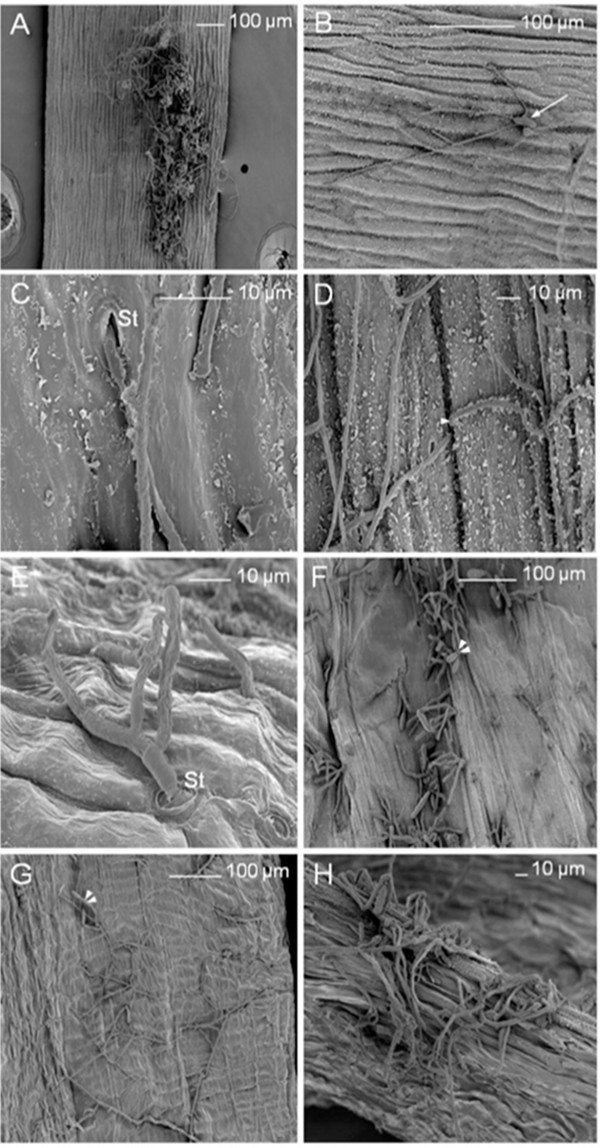
***A. brassicicola* localization on *A. thaliana* siliques.** Scanning electron microscopy images of *Arabidopsis thaliana* (L*er* ecotype) siliques 10 days after inoculation with *Alternaria brassicicola* (Abra43). (**a**), mycelium development on the outer surface of a silique in many directions from the inoculation point. (**b**), conidia germination (white arrow) on the outer surface of a silique. (**c**), hyphal penetration into silique tissues via a stomatum (St). (**d**), direct hyphal penetration into silique tissues at cellular junctions (white arrowhead). (**e**), hyphal reemergence from silique tissues via a stomatum (St). (**f**), conidia formation (double white arrowheads) on the outer surface of a silique. (**g**), mycelium development and conidia formation (double white arrowheads) on the inner surface of a silique. (**h**), abundant fungal colonization of the replum.

On the outer surface of siliques, mycelium was found to be growing in many directions from germinated conidia in the inoculation zone (Figure 6a and 6b). Hyphae penetrated into the fruit occasionally via stomata present on the outer surface of siliques (Figure 6c) or, more often directly, in particular at the cellular junctions (Figure 6d). Stomata also allowed mycelium reemergence (Figure 6e). Conidia formation was evident on the outer surface of siliques (Figure 6f). Intense mycelium development and conidia formation were also visible on the inner surface of siliques (Figure 6g). Replum seemed to be a preferential zone for penetration into siliques (Figure 6h). The silique results indicated that the fungal pathogen spread into the seed from the fruit.

### Influence of fungal and host genotypes on seed transmission

In order to confirm that the above-described model pathosystem is a useful tool for analyzing the molecular factors involved in the seed transmission process, the seed transmission capacity of an osmosensitive fungal mutant *nik1Δ3*[18] and its wild-type parental strain (Abra43) were assessed on an *A. thaliana* L*er* ecotype. This was performed as described above by inoculating the five oldest siliques on plants on which 10 siliques were present on the main flowering stem, and individually harvesting seeds 10 days after inoculation. Parallel leaf inoculation experiments were conducted to ensure that the *nik1Δ3* mutant strain was still virulent on *A. thaliana* vegetative organs (Additional file 1).

**Figure 7:**
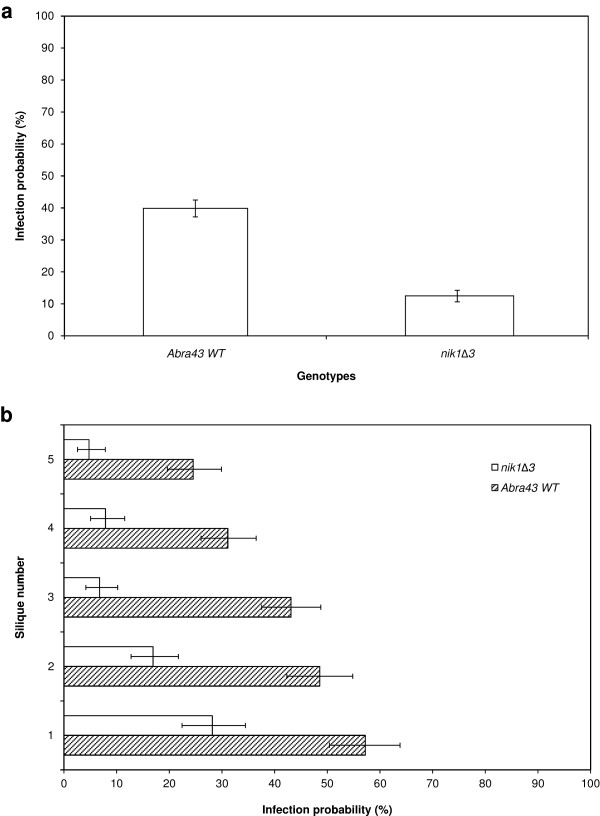
**Influence of *A. brassicicola* genotypes on seed transmission capacity.** Transmission capacity of two *Alternaria brassicicola* genotypes, Abra43 and *nik1Δ3,* to *Arabidopsis thaliana* seeds (L*er* ecotype). (**a**), global seed transmission capacity. (**b**), seed transmission capacity according to the silique stage. Hatched bars: Abra43, white bars: *nik1Δ3*. Harvesting was done 10 days after inoculation. Values represent means with IC_95._

In comparison to the wild-type strain Abra43, the *nik1Δ3* mutant strain, defective in *AbNIK1*, encoding a group III osmosensor histidine kinase, was significantly affected with respect to the transmission capacity to L*er* ecotype seeds (Figure 7). Indeed, infection probability was 40% after inoculation with Abra43, and 12% after inoculation with the *nik1Δ3* mutant strain (Figure 7a). This difference was statistically significant overall (*p* < 2 × 10^-16^), and for each silique (*p* ≤ 1.14 × 10^-8^) (Figure 7b). The gradient of fungal incidence from the oldest silique (n°1) to the youngest inoculated one (n°5) observed after inoculation with Abra43 was also noted for the *nik1Δ3* mutant strain.

**Figure 8:**
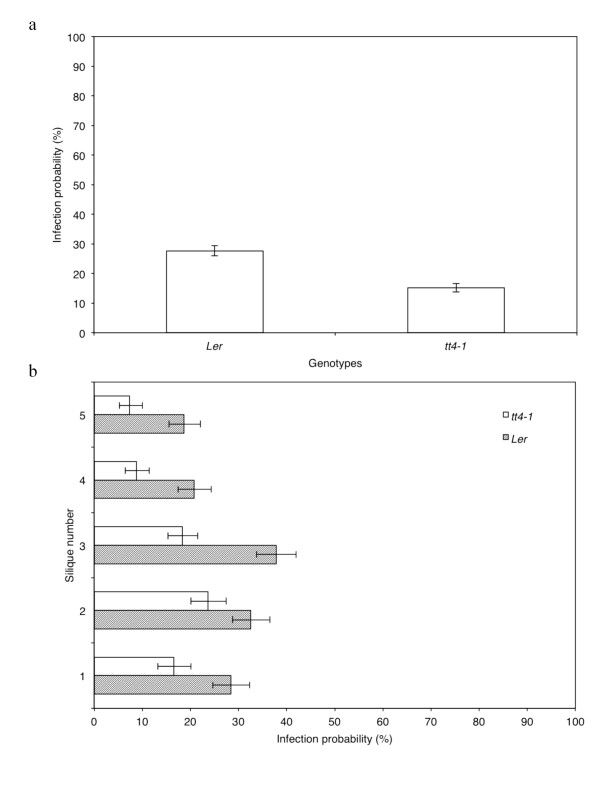
**Influence of* A. thaliana* genotypes on seed transmission capacity.** Transmission capacity of *Alternaria brassicicola* (Abra43) to seeds of two *Arabidopsis thaliana* genotypes: L*er* ecotype and *tt4-1* mutant. (**a**), global seed transmission capacity. (**b**), seed transmission capacity according to silique stage. Hatched bars: L*er* ecotype, white bars: *tt4-1* mutant. Harvesting was done 10 days after inoculation. Values represent means with IC_95._

Seed transmission efficiencies of *A. brassicicola* (Abra43) on two *A. thaliana* genotypes, the L*er* ecotype and the *tt4-1* mutant, inactivated in flavonoid biosynthesis, were also compared. Unexpectedly, the infection probability of *A. brassicicola* to *tt4-1* seeds was significantly lower (15%) than to seeds of the parental wild-type L*er* ecotype (27%) (Figure 8a). The difference in the infection probability was significant overall (*p* < 2 × 10^-16^), and for each inoculated silique (*p* ≤ 12 × 10^-4^) (Figure 8b). Parallel leaf inoculation assays did not reveal any obvious difference in the capacity of *A. brassicicola* to develop symptoms on vegetative organs from either *A. thaliana* genotypes (Additional file 1).

## Discussion

The main purpose of this study was to set up a new pathosystem that could be used to analyze molecular factors that mediate the transmission of fungal pathogens to seeds. *Arabidopsis thaliana* reproductive organs were inoculated with the seedborne pathogen *A. brassicicola*. Some general conclusions can be drawn from the findings of experiments designed to define the optimal conditions for studying fungal transmission to seeds of *A. thaliana*. First, concerning the seed transmission routes, our results did not reveal whether *A. brassicicola* was able to cause seed infection via flowers, since floral development was interrupted due to inoculation with a virulent isolate. Oliver et al. [2] demonstrated, via glasshouse inoculation trials and examination of naturally infected *Cakile* seeds, that seed infection could occur through the flowers, though less efficiently than the fruit route. Indeed, the floral route seems to be more specific to opportunistic pathogenic strains of *Alternaria alternata*, with this phenomenon being explained by the weakness of flower defence mechanisms. By contrast, we found that the fruit route was efficient and the seed contamination levels (30%) thus observed in *A. thaliana* under our experimental conditions were comparable to those obtained by Oliver et al. [2] for *Cakile maritima* seeds in field and laboratory conditions, with the frequency of infection positively correlated with the lesion density on the fruit surface.

Our results also confirmed data obtained on *Brassica* seed crops in field conditions, indicating a vertical disease gradient on fruits, with the infection first affecting the lowest siliques and then spreading slowly upwards [14]. This was explained by the observation that conidia are often disseminated short distances and retained within the canopy. As in our laboratory conditions, inoculum was distributed equally on all siliques, the more efficient transmission to seeds from the lower siliques could be mainly due to silique maturity differences, as *Alternaria* development is favored on senescent tissues [19].

The mode of seed infection by *A. brassicicola* and its localization on the reproductive organ tissues was already described several years ago [15]. We used our model pathosystem to perform a microscopic analysis of contaminated seeds, but also of contaminated siliques. Concerning seeds, surface colonization via conidia and hyphae was observed and resulted in desquamation of the seed surface, in accordance with previously published data [15]. Such intensive fungal development could be favored by the release of carbohydrates at the surface of damaged seeds as proposed by Knox-Davies [15]. Conidiogenesis has frequently been observed on contaminated seeds, with spores preferentially located in seed coat folds and cracks, which may provide protection against harsh environments [20]. Superficial hyphae also penetrated within the seed, as confirmed by SEM and confocal laser scanning microscopy. Our observations did not allow us to determine if *A. brassicicola* was limited to the seed coat or also present inside the endosperm and embryo. However, according to Singh & Mathur [17], whereas biotrophic fungi are often located in the embryo, necrotrophic fungi like *A. brassicicola* are generally located on seed and fruit coats, and deeper penetration is uncommon. In line with this, *A. brassicicola* was found to be confined to the testa of naturally infected Brussels sprout seeds while being mainly on the surface cabbage seeds [15]. The extent of testa colonization could not be accurately determined and would require transmission electronic microscopy analysis of ultra-thin seed cuts or confocal laser scanning microscopy analysis of seed cuts. Nevertheless, hyphal penetration of *A. thaliana* seed coats was evidently accompanied by seed surface damage. This phenomenon could be due to the production of plant tissue degradation enzymes, for example cutinases, as already described for *A. brassicicola*, and it could potentially accompany the penetration of cabbage tissues [21,22]. The same hypothesis was put forward by Vaughan et al. [23] with respect to soybean seed colonization by several *Alternaria* sp.. To our knowledge, no detailed microscopic analysis has ever been reported concerning silique colonization. A few days before seed harvest, typical black-spot symptoms developed on fruits at inoculation sites where intensive development of the fungus was observed on the outer surface of siliques. Penetration inside the fruit was either via stomata, intercellular spaces or replum, with occasional differentiation of appressoria-like structures.

The model pathosystem thus defined was then used to specify the molecular factors impacting the seed colonization process by using mutant genotypes of the pathogen and host plant. In maturing seeds, the water content dramatically decreases, leading to a reduction in the metabolism necessary for seed conservation. During seed colonization, fungi could thus be exposed to severe water and osmotic stresses that they have to overcome to be efficiently transmitted to seeds and complete their infection cycle. Then the ability to cope with water and osmotic stress could be a factor that determines their transmission to seeds. *Arabidopsis thaliana* silique inoculation in controlled conditions allowed us to repeatedly show a lower seed transmission capacity for *nik1Δ3*, a disruption mutant deficient in a group III histidine kinase involved in the osmotic stress response [18], by comparison with its wild-type parental strain Abra43. The contamination level was significantly lower at every silique stage, with a gradient from the oldest to the youngest silique, as observed after Abra43 inoculation. This confirms previous findings indicating that *A. brassicicola* null mutants in the *AbNIK1* gene, although their virulence on host vegetative tissues remained intact, were affected in their transmission to radish seeds after artificial inoculation in field conditions [7].

Using our model pathosystem, we took advantage of the availability of *A. thaliana transparent testa (tt)* mutants, to assess the influence of flavonoids present in seed testa on their susceptibility to fungal infection. The selected mutant line *tt4* is altered in the chalcone synthase gene [24] and therefore disrupted in the first step of the flavonoid biosynthesis pathway. It produces pale yellow seeds and does not produce any of the flavonoid compounds present in WT seeds, i.e. proanthocyanidins and flavonols [25]. Based on the well-documented plant protection functions of flavonoids against abiotic and biotic stresses [26], and previously published data showing that peas with dark seed coats were less susceptible to fungal infections than those with light-colored coats [27], we anticipated that *A. brassicicola* would colonize seeds of the *tt4-1* allele more efficiently than the wild type. Unexpectedly, although a downward vertical gradient of seed infection was still observed for both wild-type and mutant genotypes, significantly lower *A. brassicicola* infection probabilities were obtained with the flavonoid deficient mutant. A recent metabolomic study characterized several compounds over-accumulating in seeds of *tt4-1* plants compared to those of wild-type L*er*, as phenolic choline esters, sinapate-derived metabolites and glucosinolate breakdown products [28]. Although the antimicrobial properties of the former compounds have yet to be characterized, glucosinolate derivatives have been well documented as potentially toxic for pathogens, including *A. brassicicola *[29], and such metabolomic changes in *tt4-1* seeds might explain our observations. These hypotheses can be tested by investigating the transmission behaviour of *Arabidopsis* mutants disrupted in sinapate and glucosinolate metabolisms. Complementary investigations using different *tt* mutants and different *tt4* alleles are required to reliably conclude on the role of flavonoids in the seed transmission process of *A. brassicicola* to *A. thaliana.*

## Conclusions

We have established a reliable *Arabidopsis-*based pathosystem allowing investigations of fungal transmission to seeds. The results obtained by comparing the seed transmission ability of two fungal genotypes (wild-type vs. *nik1Δ3*) on the L*er* ecotype and of the wild-type isolate Abra43 on two plant genotypes (L*er* vs. *tt4-1*) confirmed that the described assay was suitable for assessing the impact of different fungal or plant mutations on the seed transmission process. This new pathosystem therefore paves the way towards future studies involving other fungal genotypes, e.g. hypersensitive to osmotic and/or water stresses, and other plant genotypes, e.g. mutants producing abnormal seed coats. Moreover, fungus-infected seeds can now be reproducibly obtained and used as starting material to study the ability of the pathogen to survive in seeds during storage and to transmit from seeds to seedlings.

## Methods

### Fungal strains and growth conditions

The *A. brassicicola* strains Abra43 (wild-type) and *nik1Δ3* (mutant inactivated in the *AbNIK1* gene) used in this study have previously been described [18,29]*.* For routine culture, Abra43 and *nik1Δ3* were grown and maintained on potato dextrose agar (PDA) and PDA supplemented with 12 μg/mL hygromycin B, respectively.

### *Arabidopsis thaliana* genotypes and growth conditions

The capacity of *Alternaria brassicicola* to transmit to seeds was studied in plants obtained by sowing seeds of *Arabidopsis thaliana* individually in pots (7 x 7 x 7 cm) containing prewetted compost (NEUHAUS HUMINSUBSTRAT *N4*, NFU 44–551). The Landsberg *erecta* (L*er*) ecotype and its *tt4-1* mutant allele (NASC reference N85), inactivated in the chalcone synthase gene and totally defective in flavonoids [24,30], were used. The plants were grown in a controlled climatic room with day/night temperatures of 22°C/19°C and a 16 h photoperiod. ARACON base and tube (ARASYSTEM, BETATECH bvba, Gent, Belgium) were placed as soon as the floral stem appeared.

### Infection assays and seed contamination assessment

The floral infection route for seed transmission was tested by inoculating *A. thaliana* floral buds with 5 μL drops of an *A. brassicicola* conidial suspension. Two parallel experiments were performed with conidial suspension adjusted to either 1 x 10^4^ or 1 x 10^5^ conidia mL^-1^. The plants were maintained under saturating humidity for 2 days in the dark and were monitored 6 and 14 days after inoculation.

Most of the seed transmission assays used 1-month-old *A. thaliana* plants with 10 (or 20 for determination of the influence of the plant developmental stage on seed transmission) siliques. The position of a silique at a precise stage of development is determined by the number of flowers produced per inflorescence per day, which is in general three or four. Two 2,5 μL drops of an *A. brassicicola* conidial suspension (1 x 10^5^ conidia mL^-1^ in water) supplemented with 0,01% (v/v) Tween 20 were placed on the 10 (or 20 for determination of the influence of the plant developmental stage on seed transmission) youngest siliques (one drop at the silique base and one in the middle). The oldest silique (at the base of the bolting stem) was numbered 1 and the youngest 10 (or 20 for determination of the influence of the plant developmental stage on seed transmission). At least three plants per fungal or plant genotype were inoculated and the experiment was repeated twice. As a control for all experiments, two 2,5 μL drops of a 0,01% (v/v) Tween 20 solution were placed on 10 siliques of one plant. The plants were then maintained under saturating humidity for 2 days in the dark.

Contaminated siliques were harvested 10 days after inoculation (or 20 days after inoculation for determination of the influence of the harvest date on seed transmission). Inoculated or control siliques were dissected with sterile forceps and seeds were carefully harvested to avoid contact with the fungus potentially present on the outer surface of siliques. Seeds were incubated separately on PDA medium for 2 days. A seed was considered to be contaminated when incubation resulted in typical colony development. As the first results showed that contamination levels were very low for siliques n°6 to n°10, only results for siliques n°1 to n°5 were taken into account for further experiments.

### Statistical analysis

Because of their dichotomous nature (i.e. each seed is either contaminated or not), seed contamination data were analyzed using logistic (logit) generalized linear models (for an introduction, see [31]). Statistical analyses were performed with the R software package (version 2.12.0, [32]), using the glm function with “family” set to “binomial (link = “logit”)”. IC_95_ (95% confidence interval) values were extracted from such models. For ease of reading, log odd values predicted by the models were converted to infection probabilities, thus resulting in slightly asymmetric IC_95_s. Repetitions were treated as a factor, and were found not to have a significant effect.

### Scanning electronic microscopy (SEM)

For SEM observations, specimens (siliques valves and seeds harvested 10 days after inoculation) were fixed under vacuum in 4% (v/v) glutaraldehyde in 0.1 M phosphate buffer (pH 7.2) for 4 h at 4°C and stored overnight at 4°C in the same fixative buffer. Specimens were rinsed once in 0.1 M phosphate buffer (pH 7.2) and post-fixed in 2% (w/v) osmium tetroxide in 0.1 M phosphate buffer (pH 7.2) in the dark for 2 h. Samples were rinsed three times with distilled water, dehydrated in a graded ethanol series (50, 70, 95 and 100% for 10 min each). The last 100% ethanol bath was repeated three times. The dehydration process was completed by treating samples in ethanol/acetone (1:1, v/v) for 15 min and then twice in acetone for 15 min. Specimens were dried by the critical-point method. Silique walls and seeds were then sputter-coated with a thin carbon layer and examined using a JSM 6301-F (JEOL, Tokyo, Japan) scanning electron microscope operating at 3 kV.

### Confocal laser scanning microscopy

For confocal laser scanning microscopy observations, seeds harvested 10 days after inoculation were observed after double staining: seed cell walls were stained with propidium iodide [33] and fungal cell walls, with solophenyl flavin [34].

Seeds were immersed for 2 min in a 1% (w/v) propidium iodide solution and rinsed twice in 0.1 M phosphate buffer (pH 7.2). Then the seeds were incubated in a 0.1% (w/v) solophenyl flavin solution for 5 min and rinsed twice in ultrapure water, and then stored in 50% (v/v) glycerol at 4°C until analysis under a Nikon (Nikon Instruments, Melville, NY) A1S1 confocal laser microscope equipped with argon-ion (488 nm) and diode (561 nm) lasers. Solophenyl flavin staining was detected with the emission light set at 520 nm, and propidium iodide staining with the emission light set at 617 nm. Several images were overlapped to obtain a tridimensional picture.

## Competing interests

The authors declare that they have no competing interests.

## Authors’ contributions

SP developed the seed transmission pathosystem, performed the experimental work and wrote a draft of the manuscript. ET prepared most of the plant samples for microscopy. BI carried out initial experiments aimed at studying the seed transmission routes. TG and IB supervised experiments involving *A. brassicicola* and *A. thaliana* mutants, respectively. SG and MJ provided technical assistance with scanning electronic and confocal laser scanning microscopy, respectively. RB performed the statistical analyses. PS and CC designed and coordinated the project and wrote the manuscript. All authors performed a critical reading and approved the final version of the manuscript.

## Supplementary Material

Additional file 1: **Effects of the disruption of *AbNIK1 *on the virulence of *A. brassicicola *on *A. thaliana *and of the *tt4 *mutation on the susceptibility of *A. thaliana *to *A. brassicicola.*** Leaves of *A. thaliana* L*er* ecotype or its *tt4 mutant* were inoculated with conidia suspensions of Abra43 and *nik1Δ3* strains. For each plant-pathogen combination, five leaves are shown that correspond to representative symptoms obtained at 6 dpi. Click here for file

## References

[B1] Van den Bosch F Fraaije BA van den Berg F Shaw MW Evolutionary bi-stability in pathogen transmission mode Proc Royal Soc B 2010 277 1735 1742 10.1098/rspb.2009.2211 PMC2871859 20129975

[B2] Oliver EJ Thrall PH Burdon JJ Ash JE Vertical disease transmission in the Cakile-Alternaria host-pathogen interaction Aust J Bot 2001 49 561 569 10.1071/BT00068

[B3] Hewett PD Septoria nodorum on seedlings and stubble of winter wheat Trans Br Mycol Soc 1975 65 7 18 10.1016/S0007-1536(75)80176-8

[B4] Baker KF Smith SH Dynamics of seed transmission of plant pathogens Annu Rev Phytopathol 1966 14 311 332

[B5] Elmer WH Seeds as vehicles for pathogen importation Biol Invasions 2001 3 263 271 10.1023/A:1015217308477

[B6] Cappelli C Seeds: pathogen transmission through Encyclopedia of Plant and Crop Science. Boca Raton 2007 Taylor & Francis, Florida 1142 1147

[B7] Iacomi-Vasilescu B Bataillé-Simoneau N Campion C Dongo A Laurent E Serandat I Hamon B Simoneau P Effect of null mutations in the AbNIK1 gene on saprophytic and parasitic fitness of Alternaria brassicicola isolates highly resistant to dicarboximides fungicides Plant Pathol 2008 57 937 947 10.1111/j.1365-3059.2008.01864.x

[B8] Schlaich NL Arabidopsis thaliana: the model plant to study host-pathogen interactions Curr Drug Targets 2011 12 955 966 10.2174/138945011795677863 21366521

[B9] Lawrence CB Mitchell TK Craven KD Cho Y Cramer RA Kim KH At death’s door: Alternaria pathogenicity mechanisms Plant Pathol J 2008 24 101 111 10.5423/PPJ.2008.24.2.101

[B10] Maude RB Humpherson-Jones FM Studies on the seed-borne phases of dark leaf spot (Alternaria brassicicola) and grey leaf spot (Alternaria brassicae) of brassicas Ann Appl Biol 1980 95 311 319 10.1111/j.1744-7348.1980.tb04752.x

[B11] Maude RB Humpherson-Jones FM Shuring CG Treatments to control Phoma and Alternaria infections of brassica seeds Plant Pathol 1984 33 525 535 10.1111/j.1365-3059.1984.tb02877.x

[B12] Humpherson-Jones FM The incidence of Alternaria spp. and Leptosphaeria maculans in commercial brassica seed in the United Kingdom Plant Pathology 1985 34 385 390 10.1111/j.1365-3059.1985.tb01377.x

[B13] Kubota M Abiko K Yanagisawa Y Nishi K Frequency of Alternaria brassicicola in commercial cabbage seeds in Japan J Gen Plant Pathol 2006 72 197 204 10.1007/s10327-006-0272-1

[B14] Humpherson-Jones FM Maude RB Studies on the epidemiology of Alternaria brassicicola in Brassica oleracea seed production crops Ann Appl Biol 1982 100 61 71 10.1111/j.1744-7348.1982.tb07192.x

[B15] Knox-Davies PS Relationships between Alternaria brassicicola and Brassica seeds Trans Br Mycol Soc 1979 73 235 248 10.1016/S0007-1536(79)80107-2

[B16] Maude RB Seedborne diseases and their control: principles and practice 1996 CAB International, Wallingford, United Kingdom

[B17] Singh D Mathur SB: Histopathology of seed-borne infections 2004 CRC Press LLC, Boca Raton, Florida

[B18] Dongo A Bataillé-Simoneau N Campion C Guillemette T Hamon B Iacomi-Vasilescu B Katz L Simoneau P The group III two-component histidine kinase of filamentous fungi is involved in the fungicidal activity of the bacterial polyketide ambruticin Appl Environ Microbiol 2009 75 127 134 10.1128/AEM.00993-08 19011080 PMC2612220

[B19] Thomma BPHJ Alternaria spp.: from general saprophyte to specific parasite Molecular Plant Pathology 2003 4 225 236 10.1046/j.1364-3703.2003.00173.x 20569383

[B20] Köhl J van der Wolf JM Alternaria brassicicola and Xanthomonas campestris pv. campestris in organic seed production of Brassicae: epidemiology and seed infection Wageningen: Plant Research International 2005 Note 363, [http://edepot.wur.nl/17130]

[B21] Fan CY Köller W Diversity of cutinases from plant pathogenic fungi: differential and sequential expression of cutinolytic esterases by Alternaria brassicicola FEMS Microbiol Lett 1998 158 33 38 10.1111/j.1574-6968.1998.tb12796.x

[B22] Srivastava A Ohm RA Oxiles L Brooks F Lawrence CB Grigoriev IV Cho Y A zinc finger family transcription factor AbVf19 is required for the induction of a gene subset important for virulence in Alternaria brassicicola Mol Plant-Microbe Interact 2012 25 443 452 10.1094/MPMI-10-11-0275 22185468

[B23] Vaughan DA Kunwar IK Sinclair JB Bernard RL Routes of entry of Alternaria sp. into soybean seed coats Seed Science and Technology 1988 16 725 731

[B24] Shirley BW Kubasek WL Storz G Bruggemann E Koornneef M Ausubel FM Goodman HM Analysis of Arabidopsis mutants deficient in flavonoid biosynthesis Plant Journal 1995 8 659 671 10.1046/j.1365-313X.1995.08050659.x 8528278

[B25] Routaboul JM Kerhoas L Debeaujon I Pourcel L Caboche M Einhorn J Lepiniec L Flavonoid diversity and biosynthesis in seed of Arabidopsis thaliana Planta 2006 224 96 107 10.1007/s00425-005-0197-5 16395586

[B26] Pourcel L Routaboul JM Cheynier V Lepiniec L Debeaujon I Flavonoid oxidation in plants: from biochemical properties to physiological functions Trends Plant Sci 2007 12 29 36 10.1016/j.tplants.2006.11.006 17161643

[B27] Mohamed-Yaseen Y Barringer SA Splittstoesser WE Costanza S The role of seed coats in seed viability Bot Rev 1994 60 246 260

[B28] Böttcher C von Roepenack-Lahaye E Schmidt J Schmotz C Neumann S Scheel D Clemens S Metabolome analysis of biosynthetic mutants reveals a diversity of metabolic changes and allows identification of a large number of new compounds in Arabidopsis Plant Physiol 2008 147 2107 2120 10.1104/pp.108.117754 18552234 PMC2492617

[B29] Sellam A Iacomi-Vasilescu B Hudhomme P Simoneau P In vitro antifungal activity of brassinin, camalexin and two isothiocyanates against the crucifer pathogens Alternaria brassicicola and Alternaria brassicae Plant Pathol 2007 56 296 301 10.1111/j.1365-3059.2006.01497.x

[B30] Debeaujon I Léon-Kloosterziel KM Koornneef M Influence of the testa on seed dormancy, germination, and longevity in Arabidopsis Plant Physiol 2000 122 403 413 10.1104/pp.122.2.403 10677433 PMC58877

[B31] Rodríguez G Lecture Notes on Generalized Linear Models 2007 [http://data.princeton.edu/wws509/notes/]

[B32] R Development Core Team R: A language and environment for statistical computing 2010 R Foundation for Statistical Computing, Vienna

[B33] Truernit E Haseloff J A simple way to identify non-viable cells within living plant tissue using confocal microscopy Plant Methods 2008 4 15 10.1186/1746-4811-4-15 18573203 PMC2442066

[B34] Hoch HC Galvani CD Szarowski DH Turner JN Two new fluorescent dyes applicable for visualization of fungal cell walls Mycologia 2005 9 580 588 16392246 10.3852/mycologia.97.3.580

