# Supplementary material for: The Arabidopsis thaliana-Alternaria brassicicola pathosystem: A model interaction for investigating seed transmission of necrotrophic fungi
Source: Plant Methods. 2012 May 9;8:16. doi: 10.1186/1746-4811-8-16 (PMC3445844; doi:10.1186/1746-4811-8-16)
Supplement: Additional file 1: — Effects of the disruption of AbNIK1 on the virulence of A. brassicicola on A. thaliana and of the tt4 mutation on the susceptibility of A. thaliana to A. brassicicola. Leaves of A. thaliana Ler ecotype or its tt4 mutant were inoculated with conidia suspensions of Abra43 and nik1Δ3 strains. For each plant-pathogen combination, five leaves are shown that correspond to representative symptoms obtained at 6 dpi. [file 1746-4811-8-16-S1.pptx]

## Slide 1
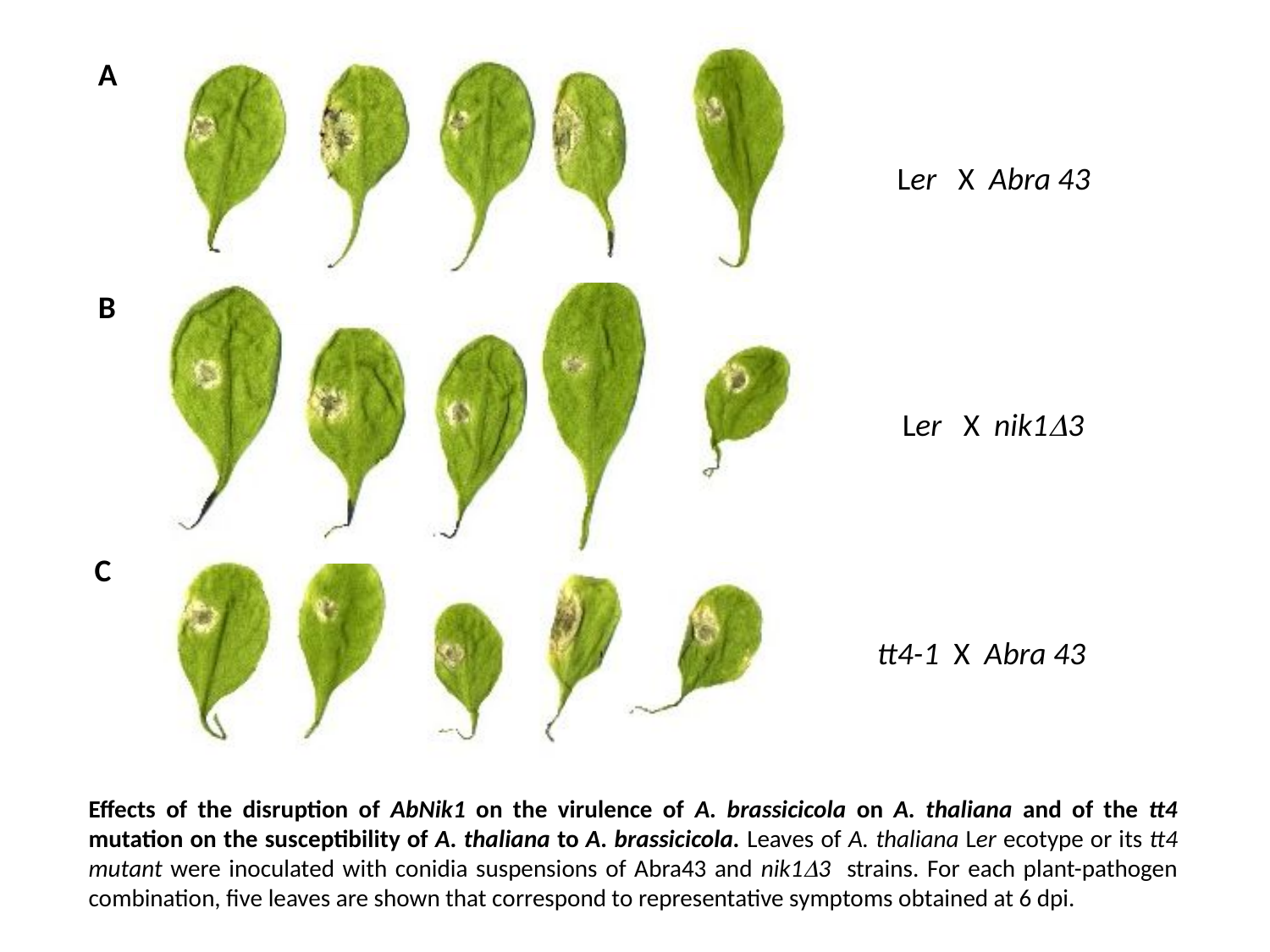

A
Ler X Abra 43
B
Ler X nik1D3
C
tt4-1 X Abra 43
Effects of the disruption of AbNik1 on the virulence of A. brassicicola on A. thaliana and of the tt4 mutation on the susceptibility of A. thaliana to A. brassicicola. Leaves of A. thaliana Ler ecotype or its tt4 mutant were inoculated with conidia suspensions of Abra43 and nik1D3 strains. For each plant-pathogen combination, five leaves are shown that correspond to representative symptoms obtained at 6 dpi.
